# Improvement of residual dizziness associated with a low-dose vitamin D-calcium combination (Caltrate D) supplementation in female patients with benign paroxysmal positional vertigo after successful repositioning: an 8-week retrospective study

**DOI:** 10.3389/fnut.2026.1928365

**Published:** 2026-09-18

**Authors:** Weiwei Han, Ange Li, Zhenyi Fan, Qiongfeng Guan, Xiaoxia Liu, Weinv Fan, Yunqin Wu

**Affiliations:** 1 Department of Rehabilitation, Ningbo No. 2 Hospital, Wenzhou Medical University, Ningbo, Zhejiang, China; 2 Department of Neurology, Ningbo No. 2 Hospital, Wenzhou Medical University, Ningbo, Zhejiang, China

**Keywords:** 25-hydroxyvitamin D, benign paroxysmal positional vertigo, Caltrate D, residual dizziness, vitamin D deficiency

## Abstract

**Background:**

Residual dizziness (RD) is a common and debilitating symptom following successful canalith repositioning procedure (CRP) for benign paroxysmal positional vertigo (BPPV). Currently, no effective pharmacological treatment exists for this condition. Our previous work identified low serum 25-hydroxyvitamin D [25(OH)D] as an independent risk factor for RD. This study therefore evaluated whether 8-week supplementation with Caltrate D was associated with improvement in RD.

**Methods:**

Female patients with primary BPPV who achieved successful CRP but still complained of RD at day 7 post-CRP and had serum 25(OH)D < 20 ng/mL were enrolled. Patients who took Caltrate D for 8 weeks formed the treatment group, while those who received no vitamin D or calcium supplementation served as the control group. The primary outcome was the improvement rate of the Dizziness Handicap Inventory (DHI) total score at 4 and 8 weeks. Secondary outcomes included duration of RD.

**Results:**

Forty-three patients in the treatment group and 48 in the control group completed the study. At 4 and 8 weeks, DHI improvement rates were significantly higher in the treatment group than in the control group (56.2% ± 10.8% vs. 44.4% ± 11.8 and 77.6% ± 6.34% vs. 66.9% ± 9.8%, respectively; both *p* < 0.001). The median RD duration was also significantly shorter in the treatment group (12 days, IQR 7–18 vs. 19 days, IQR 12–24; *p* = 0.007) than in the control group.

**Conclusion:**

In this retrospective cohort of female BPPV patients with RD and vitamin D deficiency after successful CRP, 8-week Caltrate D supplementation was associated with improvement in DHI score and shortening of RD duration. Prospective studies are warranted to confirm these observations.

## Introduction

Benign paroxysmal positional vertigo (BPPV) is the most common peripheral vestibular disorder, characterized by brief episodes of vertigo triggered by head position changes (1). Of note, BPPV predominantly involves the posterior semicircular canals, which are the most gravity-sensitive structures of the inner ear (2). Canalith repositioning procedure (CRP) achieves successful resolution of positional nystagmus and vertigo in 80 to 95% of patients (3). However, 30 to 60% of successfully treated patients continue to experience residual dizziness (RD), described as non-rotatory lightheadedness, unsteadiness, or a floating sensation that can persist for weeks to months and significantly impair quality of life (4, 5). The underlying mechanisms of RD remain unclear. Proposed mechanisms include persistent otolithic dysfunction, central maladaptation, psychiatric comorbidities such as anxiety and depression (6), and possibly residual microscopic otoconial debris that does not trigger nystagmus (7, 8).

Vitamin D plays a key role in calcium homeostasis and otoconial metabolism, and its deficiency has been linked to BPPV recurrence (9, 10). Several trials have confirmed that vitamin D supplementation reduces the recurrence of BPPV (11, 12, 13). Our group previously reported that low serum 25-hydroxyvitamin D [25(OH)D] level was independently associated with RD after successful CRP (14). Recently, Zhou et al. (15) found that vitamin D supplementation not only relieved symptoms but also shortened ocular vestibular evoked myogenic potential N10 latency, suggesting improved otolith function. Collectively, these findings suggest that vitamin D repletion represents a potential therapeutic strategy for RD.

Nevertheless, no study has specifically examined Caltrate D (a widely available, low-dose, over-the-counter vitamin D-calcium combination) for RD after successful CRP. Most existing interventional studies have used higher vitamin D doses and focused on recurrence prevention rather than RD (11, 16, 17, 18). Given our prior observation linking low vitamin D to RD and the emerging mechanistic support, we conducted this retrospective cohort study to evaluate whether 8-week Caltrate D supplementation relates to improvement in RD in vitamin D deficient patients with BPPV.

## Materials and methods

### Study design and ethical approval

This single-center retrospective study was conducted at the Department of Neurology, Ningbo No.2 Hospital from June 2020 to May 2025. The protocol was approved by the institutional review board (approval numbers: KY-2020-038, KY-2023-199 and KY-2026-240). Informed consent for blood sampling had been obtained from all patients as part of routine clinical care, and the requirement for additional informed consent was waived due to the retrospective nature of the study.

### Participants

Inclusion criteria were: (1) diagnosis of BPPV was established according to the Bárány Society diagnostic criteria (19), which require: (a) recurrent episodes of positional vertigo or dizziness provoked by lying down or turning over in the supine position; (b) characteristic positional nystagmus elicited by positional maneuvers (Dix–Hallpike test for posterior canal involvement, or supine roll test for horizontal canal involvement), with short latency and typical vector; (c) positional nystagmus lasting <1 min for canalolithiasis (or >1 min for cupulolithiasis); and (d) symptoms not attributable to another disorder; (2) successful CRP confirmed by negative supine roll test and Dix-Hallpike test immediately after the maneuver and confirmed again at 1 h post-procedure; (3) persistent complaint of dizziness, unsteadiness or floating sensation at day 7 after successful CRP;(4) serum 25(OH)D level < 20 ng/mL obtained within 7 days after CRP (20); and (5) age between 18 and 80 years. Given that BPPV has a higher prevalence in females than in males, we enrolled only female patients to reduce potential sex-related confounding and ensure adequate sample size.

Exclusion criteria were:(1) anterior canal BPPV or multicanal involvement; (2) secondary BPPV due to head trauma, inner ear surgery, Meniere’s disease, vestibular neuritis, or other neurological disorders; (3) persistent positional nystagmus after CRP; (4) prior use of vitamin D or calcium supplements within 3 months; and (5) conditions affecting calcium or vitamin D metabolism, including chronic kidney disease, primary hyperparathyroidism, malabsorption syndromes, use of glucocorticoids or bisphosphonates.

Exclusion criteria after enrollment (for final analysis) were: (1) adherence<80% of prescribed doses or incomplete follow-up data; (2) severe adverse events leading to discontinuation of Caltrate D supplementation; (3) receipt of other treatments during the study period.

### Exposure and grouping

All patients received standard care and were divided into two groups based on post-discharge medication records. None of the patients in either group received vestibular rehabilitation, anti-dizziness medications, or anxiolytics during the study period.

The treatment group received Caltrate D (600 mg elemental calcium + 125 IU vitamin D_3_ per tablet) once daily for 8 weeks, starting from day 7 post-CRP. Adherence (≥80%) was confirmed by pill count or medication diary.

The control group received no vitamin D or calcium supplementation during the 8-week period.

### Outcome assessment

The Dizziness Handicap Inventory (DHI) is a validated 25-item self-administered questionnaire assessing dizziness-related handicap (21). It comprises functional (9 items, 36 points), emotional (9 items, 36 points), and physical (7 items, 28 points) subscales, with a total score from 0 (no handicap) to 100 (severe handicap). The Chinese version has been validated for use in Chinese populations (22). The primary outcome was the DHI improvement rate at 4 and 8 weeks, calculated as [(baseline DHI – DHI at 4 or 8 weeks)/baseline DHI] × 100%.

Secondary outcomes included:

Duration of RD (days), defined as the number of days from successful CRP to the patient’s report of “complete disappearance of dizziness/unsteadiness” in the medical records.

Rate of patients achieving a ≥ 18-point DHI improvement at 4 and 8 weeks, defined as the minimal clinically important difference (MCID) (23).

### Data collection

The following data were extracted using a standardized data collection form.

Baseline characteristics included age, body mass index, comorbidities (hypertension, diabetes mellitus), duration of symptoms prior to treatment, number of CRP maneuvers, BPPV type, DHI total score, and serum 25(OH)D level.

Follow-up data at 4 and 8 weeks included DHI total score at both time points, duration of RD, and any documented adverse events related to Caltrate D.

### Statistical analysis

Continuous variables were expressed as mean ± standard deviation (SD) or median with interquartile range (IQR) depending on normality (tested by the Shapiro–Wilk test). Categorical variables were presented as numbers (percentages). Between-group comparisons were performed using independent-samples *t*-test or Mann–Whitney *U* test for continuous variables, and chi-square test or Fisher’s exact test for categorical variables. In addition, an analysis of covariance (ANCOVA) was performed on the 8-week DHI change score to adjust for potential confounding variables, including baseline DHI, age, BMI, BPPV type, symptom duration, CRP frequency, and baseline 25(OH)D level. All analyses were conducted using SPSS version 26.0 (IBM Corp., Armonk, NY, United States). A two-tailed *p* < 0.05 was considered statistically significant.

Post-hoc power analysis based on the observed effect size for the primary outcome (Cohen’s *d* = 1.15) indicated a power of 0.92 at *α* = 0.05, confirming sufficient sample size to detect the between-group difference.

## Results

### Patient selection and baseline characteristics

A total of 110 female patients with primary BPPV who met the inclusion criteria were initially enrolled. After grouping and excluding patients with incomplete data, 91 patients completed the 8-week study, including 43 in the treatment group and 48 in the control group. The baseline characteristics were well balanced between the two groups, with no significant differences in age, BMI, comorbidities, number of CRP maneuvers, BPPV type, baseline DHI score, or 25(OH)D level (all *p* > 0.05; Table 1; Figure 1).

**Table 1:** Baseline characteristics of the study population.

Characteristic	Treatment group (43)	Control group (48)	*p*-value
Age (years), mean ± SD	57.3 ± 11.1	54.5 ± 12.2	0.251
BMI (kg/m^2^)	23.1 ± 3.14	22.9 ± 3.08	0.776
Hypertension, *n* (%)	7 (16.3%)	9 (18.8%)	0.790*
Diabetes mellitus, *n* (%)	5 (11.6%)	7 (14.6%)	0.765*
BPPV type (posterior: horizontal)	35/8	36/12	0.462
Duration of symptoms (days), median (IQR)	4.0 (2.0-7.0)	5.0 (2.0-10.0)	0.727^#^
Number of CRP maneuvers, mean ± SD	2.21 ± 1.12	2.10 ± 1.01	0.640
Baseline DHI score, median (IQR)	32 (24, 42)	32 (24, 40)	0.88
25(OH)D (ng/mL), mean ± SD	15.16 ± 2.71	14.89 ± 2.93	0.646

Data are presented as mean ± standard deviation (SD), median (interquartile range, IQR), or number (%). BMI, body mass index; CRP, canalith repositioning procedure; DHI, dizziness handicap inventory. * Fisher’s exact test. ^#^ Mann-Whitney *U*-test. *p* < 0.05 was considered significant.

**Figure 1:**
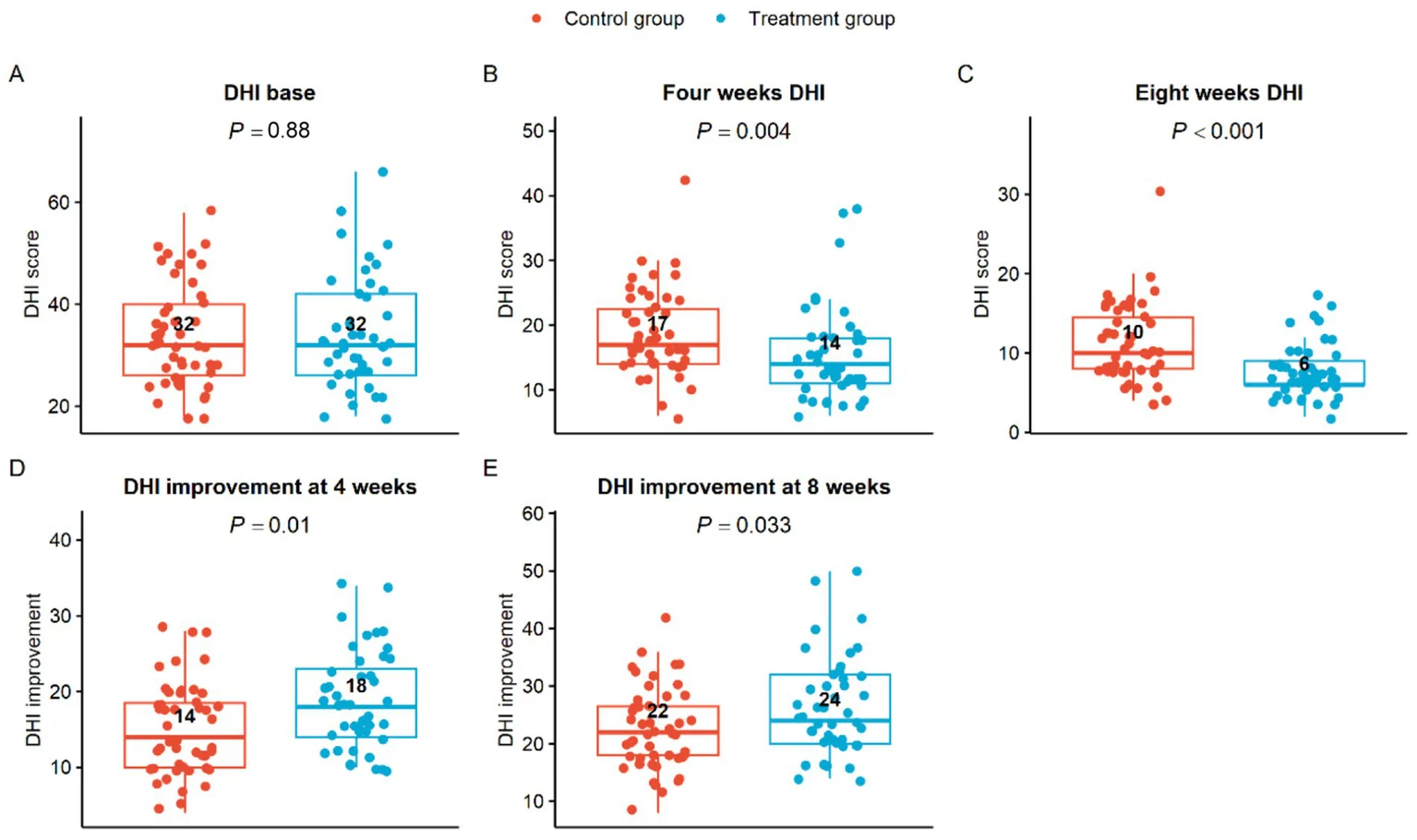
Comparison of DHI scores and improvements between the control and treatment groups. **(A)** Baseline DHI. **(B)** DHI at 4 weeks. **(C)** DHI at 8 weeks. **(D)** DHI improvement at 4 weeks, calculated as (baseline DHI – DHI at 4 weeks). **(E)** DHI improvement at 8 weeks, calculated as (baseline DHI – DHI at 8 weeks). Data are presented as median with interquartile range. *p*-values were determined using the Mann–Whitney *U*-test; *p* < 0.05 was considered significant. DHI, dizziness handicap inventory.

#### Primary outcome

At 4 and 8 weeks, the treatment group showed significantly higher improvement rates than the control group (56.2% ± 10.8% vs. 44.4% ± 11.8 and 77.6% ± 6.34% vs. 66.9% ± 9.8%, respectively, *p* < 0.001) (Table 2).

**Table 2:** Outcomes at 4 and 8 weeks.

Outcome	Treatment group (*n* = 43)	Control group (*n* = 48)	*p*-value
DHI improvement rate at 4 weeks (%), mean ± SD	56.2 ± 10.8	44.4 ± 11.8	<0.001
DHI improvement rate at 8 weeks (%), mean ± SD	77.6 ± 6.34	66.9 ± 9.8	<0.001
RD duration (days), median (IQR)	12 (7-18)	19 (12-24)	0.007
Complete resolution of RD at 8 weeks, *n* (%)	38 (88.4)	33 (68.8)	0.031
MCID achievement (≥18-point improvement of DHI) at 4 weeks, *n* (%)	24 (55.8)	22 (45.8)	0.397
MCID achievement (≥18-point improvement of DHI) at 8 weeks, *n* (%)	36 (83.7)	30 (62.5)	0.034

SD, standard deviation; RD, residual dizziness; IQR, interquartile range; DHI, dizziness handicap inventory; MCID, minimal clinically important difference. P values were calculated using an independent samples *t*-test for DHI improvement rates; Mann-Whitney *U-*test for RD duration, and chi-square test or Fisher’s exact test for categorical variables. *p* < 0.05 was considered significant.

When analyzed as absolute DHI score change, the treatment group decreased from a baseline median of 32 (IQR: 26–42) to 6 (IQR:6-10) at 8 weeks, whereas the control group decreased from a baseline median of 32 (IQR: 26–40) to 10 (IQR: 8-15.5), with the between-group comparison of change showing *p* < 0.01 (Figure 1).

### Sensitivity analysis

To further account for potential confounding, we performed an analysis of covariance (ANCOVA) on the 8-week DHI change score, with baseline DHI, age, BMI, BPPV type, symptom duration, CRP frequency, and baseline 25(OH)D level as covariates. The treatment effect remained highly significant after adjusting for these covariates (*F* = 35.79, *p* < 0.001, partial *η*^2^ = 0.30). The adjusted mean DHI change was 26.381 (95% CI: 25.51–27.26) in the treatment group vs. 22.70 (95% CI: 21.87–23.53) in the control group, with an adjusted between-group difference of 3.681 (95% CI: 2.46–4.91) in favor of Caltrate D supplementation. Baseline DHI was the strongest covariate (*F* = 521.91, *p* < 0.001), while age, BMI, CRP frequency, 25(OH)D level, and BPPV type did not significantly influence the outcome (all *p* > 0.05).

#### Secondary outcomes

The median duration of RD was significantly shorter in the treatment group (12 days, IQR: 7–18) than in the control group (19 days, IQR: 12–24, *p* = 0.007). Complete resolution of RD at 8 weeks was achieved in 38 of 43 (88.4%) patients in the treatment group, compared with 33 of 48 (68.8%) in the control group (*p* = 0.031). The proportion of patients achieving the MCID at 4 weeks was 24 of 43 (55.8%) in the treatment group vs. 22 of 48 (45.8%) in the control group (*p* = 0.397). At 8 weeks, the corresponding rate was 36 of 43 (83.7%) vs. 30 of 48 (62.5%) (*p* = 0.034) (Table 2).

## Discussion

This is the first study, to our knowledge, to examine whether correcting vitamin D deficiency with Caltrate D is associated with improvement in RD after successful CRP in BPPV. We observed that 8-week Caltrate D supplementation was significantly associated with improvement in RD: DHI improvement rate was 56.2% vs. 44.4% at 4 weeks and 77.6% vs. 66.9% at 8 weeks in the treatment group vs. the control group, and median duration of RD shortened from 19 days to 12 days.

Our previous prospective study established an association between low serum 25(OH)D and the presence of RD (14). The current study extends this finding by providing preliminary observational evidence that vitamin D repletion may alleviate RD, but causality cannot be established given the retrospective design.

Although the primary focus of this study is RD, the well-established relationship between vitamin D and BPPV recurrence provides indirect support for vitamin D’s role in otolith homeostasis. Several studies have examined vitamin D supplementation for BPPV recurrence. A 2020 randomized trial reported that vitamin D plus calcium (800 IU vitamin D_3_ plus 1,000 mg calcium daily) significantly reduced BPPV recurrence in patients with subnormal vitamin D levels (risk ratio 0.38, 95% CI: 0.21–0.68) (11). A 2025 systematic review confirmed lower vitamin D levels in patients with BPPV and reduced recurrence with supplementation (RR = 0.45, 95% CI: 0.36–0.55), although it also noted a borderline adjusted risk (RR = 0.95, 95% CI: 0.91–0.99) (9). In line with these findings, a 2025 double-blinded randomized placebo-controlled trial in older adults demonstrated that vitamin D replacement was associated with fewer recurrences and improved outcomes (18).

In contrast to recurrence-focused studies, our work addresses RD directly. Previous interventions have used higher vitamin D doses (800–2000 IU/day) over longer periods, primarily to prevent recurrence. The present study differs in three key aspects: (1) it specifically targets patients with documented vitamin D deficiency and RD;(2) it uses a fixed combination of calcium and low-dose vitamin D_3_ (125 IU/day) which is widely available over-the-counter; and (3) it suggests that 8 weeks of this regimen is sufficient to achieve symptomatic improvement in this cohort.

The underlying mechanisms may be multifactorial. Vitamin D is essential for calcium absorption and maintaining serum calcium levels. In the inner ear, vitamin D receptors are expressed in the vestibular epithelia, including the utricle and saccule (24, 25, 26). Vitamin D deficiency may lower calcium concentration in the endolymph and otoconial matrix, potentially resulting in hypomineralized and structurally fragile otoconia (8, 24). Even after successful CRP, micro-otoconia or degenerated otoconial fragments may remain attached to the otolithic membrane, causing persistent abnormal shear forces and inappropriate vestibular afferent firing, which manifests as non-rotatory dizziness (27). Restoring vitamin D could support proper mineralization of residual otoconia, reduce their mechanical irritability, and aid central vestibular compensation (15, 28).

The concurrent calcium supplementation may have contributed by suppressing parathyroid hormone-mediated bone resorption, thereby reducing the release of calcium that could otherwise destabilize otoconia. Even at the low dose of 125 IU/day, 8 weeks supplementation likely raises 25(OH)D sufficiently, especially combined with calcium, to promote otoconial stability. From a nutritional perspective, vitamin D also regulates the expression of calcium-binding proteins such as calbindin-D9k and calbindin-D28k in the inner ear, which are critical for maintaining endolymph calcium homeostasis. Vitamin D also influences muscle function and postural control through its effects on type II muscle fibers, which may indirectly contribute to the improvement of balance and dizziness symptoms. Moreover, vitamin D exhibits neurosteroid-like properties, influencing neurotrophic factors and neurotransmitter synthesis. It may enhance synaptic plasticity in the vestibular nuclei, accelerating central adaptation after peripheral insult (26, 29).

These findings have practical clinical implications. First, routine measurement of serum 25(OH)D in BPPV patients who complain of RD after successful CRP appears reasonable, given the high prevalence of vitamin D deficiency and the potential therapeutic benefit. Second, for those with confirmed deficiency, a simple 8-week course of Caltrate D may be considered as an adjunctive therapy, although these recommendations should be interpreted as preliminary pending confirmation in prospective studies. Third, the improvement over control is clinically meaningful, potentially reducing the need for additional vestibular rehabilitation or anxiolytics. It should be noted that the mechanisms underlying RD (primarily otolith dysfunction and central maladaptation) may differ from those of BPPV recurrence (otoconia regeneration and detachment). Nevertheless, both appear to benefit from vitamin D repletion.

## Limitations

This study has several limitations. First, the retrospective, non-randomized design without a placebo control introduces potential selection bias and placebo effects. Although baseline characteristics were balanced, residual confounding from unmeasured factors (e.g., psychological factors such as anxiety, depression, or somatoform disorders, dietary calcium intake, sun exposure, physical activity, socioeconomic status) cannot be excluded and may overestimate the treatment effect. Second, we did not re-measure serum 25(OH)D post-intervention, so biochemical compliance and actual vitamin D status cannot be confirmed. Additionally, the 8-week follow-up is relatively short, leaving longer-term outcomes unknown. Third, the study population was limited to female patients, which was based on the higher prevalence of BPPV in females and the need for sufficient sample size; however, the findings may not be generalizable to male BPPV patients. Fourth, the single-center design and modest sample size limit generalizability. Fifth, we did not perform objective vestibular tests (e.g., VEMPs or posturography) to quantify otolithic function, which precludes mechanistic interpretation of the observed symptom improvements. Future studies should incorporate such objective measures to elucidate the mechanisms by which vitamin D supplementation alleviates residual dizziness.

## Conclusion

In patients with BPPV who experience RD after successful canalith repositioning and have documented vitamin D deficiency, 8-week supplementation with Caltrate D was associated with significant improvement in DHI scores and shortening of RD duration in this retrospective study. Although the absence of post-treatment vitamin D measurement limits biochemical confirmation, the observed clinical benefits suggest that this simple, safe, and low-cost intervention may be a promising adjunctive therapy, but these findings should be interpreted cautiously and confirmed in future prospective studies.

## Data Availability

The raw data supporting the conclusions of this article will be made available by the authors, without undue reservation.
